# Does the Number of Turns during Sleep Have Utility in the Early Detection of Parkinson’s Disease and Its Related Disorders?

**DOI:** 10.31662/jmaj.2023-0204

**Published:** 2024-08-09

**Authors:** Tokuharu Tanaka, Hidenori Onishi, Masaki Kiyono, Yuki Miyazaki, Azusa Tanaka, Akihiko Tanizawa, Tadanori Hamano, Hiroyuki Hayashi, Koji Kobayashi, Osamu Yamamura

**Affiliations:** 1 Department of Family and Emergency Medicine, University of Fukui Hospital, Fukui, Japan; 2 Regional Medicine Promotion Course, Faculty of Medical Sciences, University of Fukui, Fukui, Japan; 3 Biomedical Imaging Research Center, University of Fukui, Fukui, Japan; 4 Sugita Genpaku Memorial Obama Municipal Hospital, Fukui, Japan; 5 Department of Neurology, University of Fukui Hospital, Fukui, Japan; 6 Department of Medical Technology, Kitasato Junior College of Health and Hygienic Sciences, Niigata, Japan

**Keywords:** Parkinson’s disease, sleep activity, number of turns, phase angle, sex-specific difference

## Abstract

**Introduction:**

Patients with Parkinson’s disease (PD) and its related disorders exhibit decreased sleep activity. However, the factors associated with this decreased sleep activity remain unknown. Thus, we aimed to explore the factors associated with sleep activity in patients with PD and its related disorders.

**Methods:**

This study included 33 patients with PD and its related disorders and 57 healthy participants who visited our outpatient clinics between November 2018 and March 2020. We evaluated the patients’ muscle masses and measured the number of times they turned during sleep. The limb skeletal muscle index was utilized to evaluate the loss of muscle mass. This study was registered in the UMIN Clinical Trials Registry (Clinical Trials Registry number: UMIN000052436).

**Results:**

Age, maximal grip strength, presarcopenia, phase angle (legs), history of hypertension, diabetes mellitus, dyslipidemia, orthopedic diseases, and the number of turns during sleep were associated with PD and its related disorders. The number of turns was independently associated with PD and its related disorders. Receiver operating characteristic curve analysis revealed that the cutoff value for the number of turns was 6 (area under the curve, 0.986; sensitivity, 93.9%; specificity, 96.5%). The cutoff numbers of turns for men and women were 9 and 6, respectively (area under the curve, 1.0 and 0.981; sensitivity, 100% and 94.7%; specificity, 100% and 95.2%; respectively).

**Conclusions:**

The number of turns during sleep is significantly associated with PD and its related disorders and may decrease before patients present with sarcopenia. In addition, PD and its related disorders may coexist in men who turn less than nine times during sleep.

## Introduction

In the early stages of Parkinson’s disease (PD) and its related disorders, patients exhibit impaired fine motor skills in the upper extremities ^(1), (2), (3), (4), (6), (7)^, which progressively hinders the execution of major activities ^(8), (9), (10), (11)^. Muscle weakness and sarcopenia, which commonly occur in patients with PD, are associated with reduced physical ability and decreased turning frequency during sleep ^(12), (13)^. Sleep disturbances are common in patients with PD and its related disorders, affecting approximately 90% of the patients ^(14)^. These disturbances include reduced sleep activity, which may decrease the quality of life and increase the risk of morbidity and mortality ^(15)^. However, only a few studies have been conducted to determine the factors associated with sleep disturbances in patients with PD.

Tossing and turning during sleep, which are associated with locomotor activity, are important indicators of sleep quality ^(16)^. Loss of muscle mass is a common feature of PD and its related disorders and is associated with decreased sleep quality ^(17)^. Furthermore, muscle strength and mass are associated with the severity of motor symptoms in PD ^(18)^. Sleep activity differs between men and women, with the former reportedly turning more often during sleep than the latter ^(19)^. Patients with PD and its related disorders exhibit decreased activity during sleep, which can be observed during the early stages of the disease. In patients with PD and its related disorders, sleep activity is often evaluated using polysomnographic and infrared cameras during sleep onset; consequently, decreased numbers of tosses and turns have been reported ^(20), (21)^. However, no study has focused on the sleep activity of patients with PD and its related disorders as well as on the early detection of these diseases.

This study aimed to evaluate sleep activity in patients with PD and its related disorders compared with healthy controls to identify factors for the early detection of the disease. It also determined whether sex-specific differences exist in sleep activity in patients with PD and its related disorders, as reported in nonpatients.

## Materials and Methods

### Study design and setting

This was a noninvasive, noninterventional, exploratory, validation, and comparative study of outpatients receiving treatment for PD and its related disorders. Eligible participants were selected from patients who visited the outpatient clinics of the University of Fukui Hospital and Sugita Genpaku Memorial Obama Hospital between April 1, 2018, and March 31, 2020. This study was approved by the Ethics Committees of the University of Fukui Hospital and Sugita Genpaku Memorial Obama Hospital (Approval No.: 20180060, 30-2). All the included participants provided written informed consent after receiving a comprehensive explanation of the study.

### Participant selection

This study included outpatients aged ≥20 years who were diagnosed with PD or its related disorders (vascular parkinsonism, progressive supranuclear palsy, dementia with Lewy bodies) and healthy participants aged 20 years or older. The participants had general medical conditions but no neurological diseases and no family history of neurological diseases.

The exclusion criteria were set to ensure the safety and well-being of potential participants, including pregnancy, potential pregnancy, lactation, or difficulty staying in general lodging hotels.

### Observational items

Patient data, including sex, date of birth, height, weight, medical and medication histories, muscle mass, grip strength, and infrared camera data (for the evaluation of sleep activity frequency), were recorded for analysis. The primary endpoint of this study was the presence of PD and its related disorders. Furthermore, sex-specific differences in the number of times the patients with PD and its related disorders turned during sleep were analyzed.

### Parkinson’s disease (PD)

According to the Movement Disorder Society’s PD criteria, parkinsonism encompasses symptoms such as bradykinesia, rest tremor, and rigidity. PD, which is a common form of parkinsonism, is a progressive disorder resulting from the degenerative loss of dopaminergic neurons. It is clinically characterized by asymmetric parkinsonism as well as clear, dramatic, and lasting effects of dopaminergic treatment. The Hoehn and Yahr (HY) severity scale is employed to indicate PD progression, particularly in the HY II stage when tremors in both limbs and bilateral muscle stiffness are observed and interference with daily life becomes apparent.

The European Working Group on Sarcopenia in the Elderly (Sarcopenia Working Group) proposed the conceptual staging of presarcopenia, sarcopenia, and severe sarcopenia ^(22)^. Presarcopenia is characterized by a loss of muscle mass without a discernible effect on muscle strength or performance. In the present study, muscle strength and physical performance were not assessed; only the presence of presarcopenia was evaluated using the skeletal muscle index (SMI), which was calculated as bilateral upper- and lower-limb fat-free mass/height^2^ (kg/m^2^). Presarcopenia was defined as SMI ≤7 and ≤5.7 kg/m^2^ for men and women, respectively ^(23)^. The limb fat-free volume was measured through bioimpedance analysis, which was conducted using a medical body composition analyzer ^(24)^.

### Phase angle

Phase angle (PhA) denotes the oscillatory disparity (phase shift) in the sinusoidal patterns of voltage and current. This is markedly observed within humans, in whom the current culminates in its maximum / minimum subsequent to the voltage (positive value) due to the prevalence of cell membranes and tissue interfaces. An augmented PhA indicates increased cellular density, structural soundness, and function. Variations in PhA values may be influenced by a range of factors, including age, sex, ethnicity, body composition, physical activity, and adiposity ^(25)^.

### Sleep activity

The participants were instructed to stay at a hotel for the sleep activity evaluation. An infrared camera was used for the evaluation. The number of turns during sleep was determined throughout the entire sleep duration. Sleep activity was classified as follows: (1) 90° rotation, 90° rotation of the shoulders and hip from the supine position or vice versa; (2) hip rotation only, 90° hip rotation (probably accompanied by leg movement) with no shoulder movement; (3) others, hand and foot movement but no shoulder or hip rotation; (4) nocturnal awakening, waking up and not being able to return to sleep; and (5) resumption of nocturnal sleep, falling asleep again during the night. The recorded images were visually assessed. All 90° rotations were recognized as sleep activities.

### Statistical analyses

Data were expressed as medians and interquartile ranges for nominal variables and as counts and percentages for categorical variables. Univariate and multivariate analyses were conducted to determine the factors associated with PD and its related disorders. Pearson’s chi-squared test, Mann-Whitney U test, and *t*-test were used for group comparisons. Moreover, multiple (binomial) logistic regression analysis was conducted to identify the factors associated with PD and its related disorders. The dependent variables substantially differed in the univariate analysis (age, PhA [legs], and number of turns). The explanatory variables were the presence of PD and its related disorders.

Statistical significance was set at *P* < 0.05. All statistical analyses were conducted using JMP version 14.2.2 (SAS Institute Inc., Cary, NC, USA).

## Results

### Participant characteristics

This study enrolled 90 participants who visited the outpatient clinics at the University of Fukui Hospital and Sugita Genpaku Memorial Obama Hospital. Of the participants, 33 (36.7%) had PD and its related disorders, including 24 with PD, 4 with vascular parkinsonism, 2 with dementia with Lewy bodies, 2 with progressive supranuclear palsy, and 1 with corticobasal degeneration, whereas 57 were healthy participants. The total sleep times of the patient and healthy groups were 442 ± 37.5 min and 444 ± 55 min, respectively. The mean age of the patient group was 73.4 ± 7.7 years, and 19 (57.6%) of the patients were women. Meanwhile, the mean age of the healthy group was 58.8 ± 12.2 years, and 42 (73.7%) of the patients were women. The mean HY stage of the patients was II. The clinical characteristics of the study participants are summarized in Table 1. The mean age of the entire study cohort was 64.2 ± 12.9 years, and 61 (67.8%) of the participants were female and 13 (14.4%) were diagnosed with presarcopenia and sarcopenia. As regards sleep activity, the mean number of turns recorded during sleep was 11.3 ± 9.4, and the mean PhA (legs) was 4.5 ± 0.8°.

**Table 1.** Background Data of the Participants.

	Total n = 90	Pt n = 33	Healthy n = 57	*P*-value
Age [years]	64.2 ± 12.9	73.4 ± 7.7	58.8 ± 12.2	<0.001
Sex [male/female]	29/61	14/19	15/42	0.180
Height [cm]	159.6 ± 9.5	159.0 ± 8.5	159.9 ± 10.1	0.798
Weight [kg]	57.9 ± 11.8	55.6 ± 10.7	59.3 ± 12.2	0.282
BMI [kg/m^2^]	22.6 ± 3.3	21.9 ± 3.7	23.0 ± 3.1	0.146
Maximum grip strength [kg]	26.1 ± 10.2	21.8 ± 8.3	28.5 ± 10.5	0.002
Muscle mass [kg; whole body]	40.6 ± 8.4	39.4 ± 7.2	41.2 ± 9.0	0.307
Muscle mass [kg; limbs]	18.0 ± 4.5	17.1 ± 3.8	18.5 ± 4.8	0.229
SMI [kg/m^2^]	6.9 ± 1.0	6.7 ± 1.1	7.1 ± 1.1	0.069
Presarcopenia	13 (14.4)	10 (30.3)	3.0 (5.3)	0.003
Bone mineral density [g/cm^2^]	2.3 ± 0.4	2.2 ± 0.3	2.4 ± 0.4	0.104
Basal metabolic rate [kcal]	1207.7 ± 237.9	1153.6 ± 191.1	1239.1 ± 257.6	0.218
Phase angle [°, legs]	4.5 ± 0.8	3.9 ± 0.6	4.9 ± 0.7	<0.001
Medical history
Hypertension	17 (18.9)	15 (45.5)	2.0 (3.5)	<0.001
Diabetes mellitus	4.0 (4.4)	4.0 (12.1)	0.0 (0.0)	0.031
Dyslipidemia	11 (12.2)	1.0 (1.8)	10 (30.3)	<0.001
Orthopedic diseases	12 (13.3)	11 (33.3)	1.0 (1.8)	<0.001
Dementia	8.0 (8.9)	8.0 (24.2)	0.0 (0.0)	<0.001
Psychotropic medications	9.0 (10.0)	4.0 (12.9)	5.0 (8.0)	0.033
Social history
Smoking	1.0 (1.1)	1.0 (3.0)	0.0 (0.0)	0.781
Alcohol consumption	3.0 (3.3)	1.0 (3.0)	2.0 (3.5)	1.000
Subjective sleep assessment
Slept well (day of the examination)	32 (35.5)	13 (39.4)	19 (33.3)	0.726
Number of turns	11.3 ± 9.4	1.7 ± 2.6	16.8 ± 7.1	<0.001

Mean ± standard deviation, number of cases (%), [unit] BMI, body mass index; SMI, skeletal muscle index Pearson’s chi-squared test, Mann-Whitney U test

### Factors associated with PD and its related disorders

**Figure 1.**
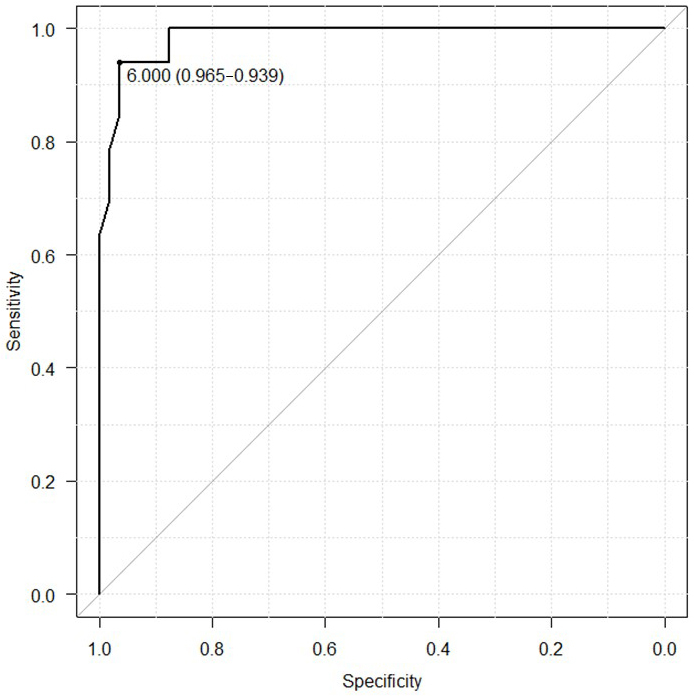
Cutoff number of turns during sleep in patients with Parkinson’s disease and its related disorders. This study included 33 patients with Parkinson’s disease and its related disorders and 57 healthy participants. The area under the curve was 0.986 (95% confidence interval, 0.969-1.000). The cutoff value was 6.0 (sensitivity, 93.9%; specificity, 96.5%).

**Figure 2.**
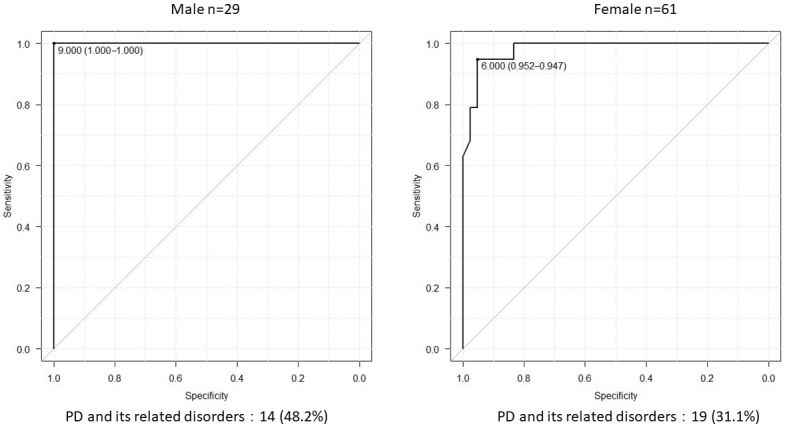
Sex-specific cutoff number of turns during sleep in patients with Parkinson’s disease and its related disorders. For men, the area under the curve was 1.0 (95% confidence interval, 1.0-1.0) and the cutoff value was 9.0 (sensitivity, 100%; specificity, 100%). For women, the area under the curve was 0.981 (95% confidence interval, 0.955-1.0) and the cutoff value was 6.0 (sensitivity, 94.7%; specificity, 95.2%).

**Figure 3.**
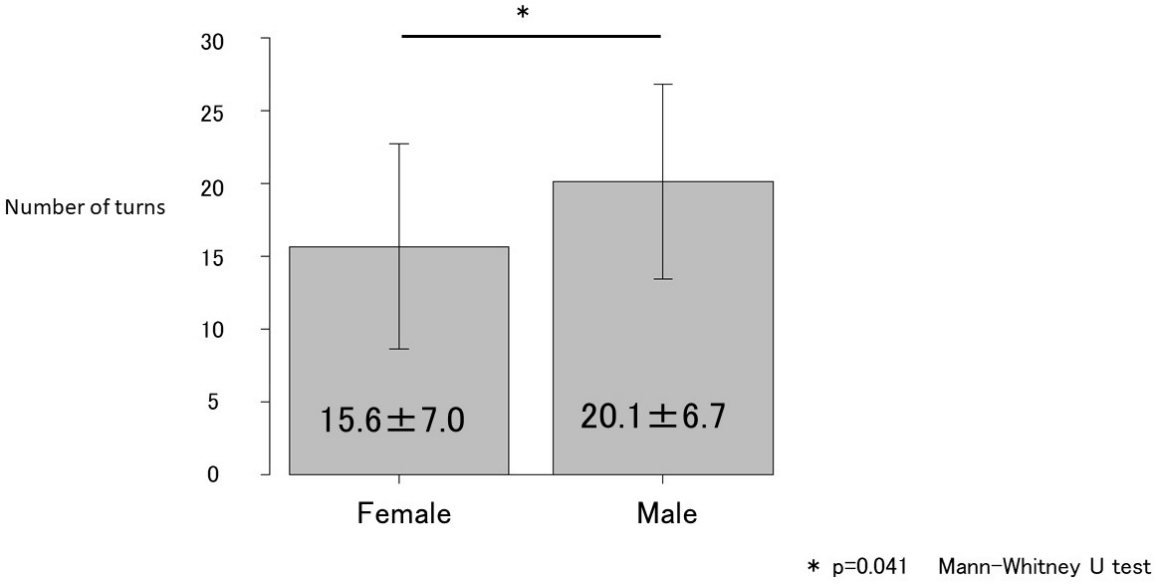
Number of turns among participants in the healthy group categorized according to sex.

Age; maximal grip strength; presarcopenia; PhA (legs); medical history of hypertension, diabetes mellitus, dyslipidemia, or orthopedic diseases; and the number of turns during sleep were associated with PD and its related disorders (Table 1). However, in the multivariate regression model, only the number of turns during sleep was independently associated with PD and its related disorders (Table 2). Receiver operating characteristic curve analysis was conducted to determine the cutoff number of turns during sleep in patients with PD and its related disorders (Figure 1). The cutoff number of turns during sleep was 6, and the area under the curve was 0.986 (sensitivity, 93.9%; specificity, 96.5%). Further analyses were conducted to examine the sex-specific differences in the cutoff numbers of turns during sleep (Table 3). The results indicated that the cutoff numbers for men and women were 9 and 6, respectively (area under the curve, 1.0 and 0.981; sensitivity, 100% and 94.7%; specificity, 100% and 95.2%; respectively; Figure 2). The sex-specific difference in the number of turns during sleep in the healthy group is shown in Figure 3.

**Table 2.** Factors Associated with Parkinson’s Disease and Its Related Disorders.

	Odds ratio	95% CI lower-upper	*p*-value
Age [years]	1.26	0.978-1.620	0.073
Phase angle [°, legs]	1.54	0.188-12.400	0.694
Number of turns	0.44	0.254-0.777	0.004

Multiple logistic regression analysis (binomial logistic regression analysis), [unit] CI, confidence interval

**Table 3.** Background Data of the Participants Categorized According to Sex.

	Male					Female			
	Total n=29	Pt n=14	Healthy n=15	*p*-value		Total n=61	Pt n=19	Healthy n=42	*p*-value
Age [years]	64.4 ± 15.3	74.5 ± 8.4	55.0 ± 14.3	0.001		64.0 ± 11.7	72.5 ± 7.3	60.1 ± 11.3	<0.001
Height [cm]	167.4 ± 8.8	165.3 ± 7.4	169.5 ± 9.8	0.149		155.8 ± 7.3	154.3 ± 6.1	156.5 ± 7.8	0.249
Weight [kg]	65.1 ± 10.6	60.8 ± 10.3	69.2 ± 9.4	0.067		54.5 ± 10.8	51.8 ± 9.4	55.7 ± 11.2	0.326
BMI [kg/m^2^]	23.2 ± 3.2	22.3 ± 4.0	24.0 ± 2.0	0.102		22.3 ± 3.4	21.7 ± 3.7	22.6 ± 3.3	0.392
Maximum grip strength [kg]	35.7 ± 11.2	28.0 ± 7.7	42.8 ± 9.2	< 0.001		21.4 ± 5.5	17.3 ± 5.3	23.3 ± 4.5	< 0.001
Muscle mass [kg; whole body]	49.0 ± 6.9	45.7 ± 5.2	52.1 ± 7.1	0.014		36.5 ± 5.6	34.7 ± 4.5	37.4 ± 5.9	0.020
Muscle mass [kg; Limbs]	22.4 ± 3.7	20.5 ± 2.5	24.1 ± 3.8	0.006		15.9 ± 3.1	14.6 ± 2.3	16.5 ± 3.2	0.012
SMI [kg/m^2^]	7.9 ± 0.9	8.4 ± 0.8	7.5 ± 0.9	0.010		6.5 ± 0.8	6.1 ± 0.8	6.7 ± 0.8	0.004
Pre-sarcopenia	5.0 (17.2)	4.0 (28.6)	1.0 (6.7)	0.285		8.0 (13.1)	6.0 (31.6)	2.0 (4.8)	0.009
Bone mineral density [g/cm^2^]	2.7 ± 0.3	2.6 ± 0.2	2.9 ± 0.4	0.022		2.2 ± 0.4	2.0 ± 0.3	2.3 ± 0.4	0.013
Basal metabolic rate [kcal]	1411.3 ± 217.9	1300.5 ± 150.8	1514.7 ± 223.9	0.016		1111.0 ± 179.6	1045.3 ± 139.0	1140.6 ± 189.2	0.031
Phase angle [°; legs]	4.8 ± 1.0	4.1 ± 0.8	5.5 ± 0.6	< 0.001		4.4 ± 0.7	3.8 ± 0.5	4.7 ± 0.6	< 0.001
Medical history
Hypertension	7.0 (24.1)	7.0 (50.0)	0.0 (0.0)	0.007		10 (16.3)	8.0 (42.1)	2.0 (4.8)	0.001
Diabetes mellitus	2.0 (6.9)	2.0 (14.3)	0.0 (0.0)	0.433		2.0 (3.2)	2.0 (10.5)	0.0 (0.0)	0.093
Dyslipidemia	5.0 (17.2)	5.0 (35.7)	0.0 (0.0)	0.040		6.0 (9.8)	5.0 (26.3)	1.0 (2.4)	0.009
Orthopedic diseases	4.0 (13.7)	4.0 (28.6)	0.0 (0.0)	0.091		8.0 (13.1)	7.0 (36.8)	1.0 (2.4)	0.001
Dementia	4.0 (13.7)	4.0 (28.6)	0.0 (0.0)	0.091		4.0 (6.5)	4.0 (21.1)	0.0 (0.0)	0.007
Social history
Smoking	0.0 (0.0)	0.0 (0.0)	0.0 (0.0)	NA		1.0 (1.6)	1.0 (5.3)	0.0 (0.0)	0.311
Alcohol consumption	1.0 (3.4)	1.0 (7.1)	0.0 (0.0)	0.483		2.0 (3.2)	0.0 (0.0)	2.0 (4.8)	1.000
Subjective sleep assessment
Slept well	11 (37.9)	4.0 (28.6)	7.0 (46.7)	0.535		21 (34.4)	9.0 (47.4)	12 (28.6)	0.244
Number of turns	11.2 ± 10.6	1.6 ± 2.6	20.1 ± 6.7	< 0.001		11.3 ± 8.8	1.7 ± 2.7	15.6 ± 7.0	<0.001

Mean ± standard deviation, number of cases (%), [unit] BMI, body mass index; NA, not available; SMI, skeletal muscle index Pearson’s chi-squared test, Mann-Whitney and t test

## Discussion

In this study, we evaluated sleep activity in patients with PD and its related disorders to identify the factors for the early detection of diseases and determine the sex-specific differences in sleep activity among patients with PD and its related disorders. To the best of our knowledge, this is the first study to analyze the factors associated with PD and its related disorders as well as the sex-specific differences in sleep activity (number of turns) in patients with PD and its related disorders. The univariate analysis revealed that age; maximal grip strength; presarcopenia; PhA (legs); medical history of hypertension, diabetes mellitus, dyslipidemia, or orthopedic diseases; and the number of turns during sleep were correlated with PD and its related disorders. Moreover, multivariate analysis revealed that the number of turns during sleep was independently associated with the presence of PD and its related disorders. Further analysis indicated that the cutoff number of turns during sleep in patients with PD and its related disorders was six. Analyses conducted according to sex revealed that the cutoff number of turns during sleep was nine for men and six for women.

PD manifests in its early stages as an impairment of upper-limb fine motor skills that subsequently progresses to impairment of larger movements. Patients with PD often exhibit sarcopenia, an age-related involuntary loss of skeletal muscle mass and strength ^(23), (26)^ and is associated with physical disability, reduced quality of life, and increased risk of death. In patients with PD and other neurodegenerative diseases, determining whether the age-related decline in physical activity is caused by disease progression or aging.

In the present study, the frequency of tossing and turning during sleep as an indicator of sleep activity was compared between the patient and healthy groups. Although numerous factors were identified in the univariate analyses, only the number of turns during sleep was associated with the presence of PD and its related disorders in the multivariate analyses. This finding indicates that the number of turns during sleep is more strongly associated with the presence of PD and its related disorders than age, muscle mass, or muscle strength. Thus, early diagnosis and treatment of PD as well as its related disorders should be considered when a patient exhibits decreased frequency of nocturnal tosses and turns. However, previous studies ^(19)^ have noted sex-specific differences in the number of times adults turn during sleep at night. Female sex, smoking, and high body mass index (BMI) were associated with less turning in bed. A negative correlation between female sex and nocturnal body movements and a positive correlation between high BMI and changes in body position were observed. Insomnia symptoms and alcohol consumption were not associated with the degree of sleep positional shifts or nocturnal body movements. In this study, no gender differences were observed in the number of smokers, BMI, or age, but grip strength and SMI tended to be higher in men. This suggests that grip strength and SMI contribute to the sex-specific differences in the number of turns during sleep. Improved nutrition and engagement in exercise therapies can help improve sleep activity. Tanaka et al. ^(27)^ reported that a Geriatric Nutritional Risk Index score ≥87 is a guideline for maintaining independent walking after 5 years. Thus, efforts aimed at achieving this goal may also be useful for preventing decreased frequency of turning during sleep.

Patients with PD tend to lose physical freedom as the disease progresses. However, no clear association was observed between the severity of PD and the frequency of turning during sleep in the present study. As regards the severity of PD, 16%, 36%, 13%, and 7% of the patients had stages I, II, III, and IV disease, respectively. Each patient was receiving treatment (levodopa) at the time of enrolment, indicating that most of them had diseases of relatively low severity. Moreover, only patients who could stay in a hotel or other similar accommodations by themselves were included. Therefore, many of the included patients had early-stage disease and were relatively responsive to pharmacotherapy. The number of participants taking psychotropic medications was 8.0% (5.0 of 57) in the healthy group and 12.9% (4.0 of 33) in the patient group. No significant difference in the prevalence of psychotropic medication use was observed between the groups. Hence, the impact of psychotropic medications on the decrease in sleep activity in the patient group was considered to be detrimental.

The findings of this study indicate that reduced sleep activity, particularly the number of turns, is associated with PD and its related disorders. Reduced frequency of turns during sleep may adversely affect the patients’ quality of sleep and overall well-being. With early diagnosis and appropriate treatment, it may be possible to maintain or improve the patients’ activities of daily living by addressing sleep-related issues. The high sensitivity and specificity of the cutoff number of turns (<6 overall, <9 for men, and <6 for women) suggest that this measure can potentially serve as an effective tool for identifying patients with PD and its related disorders. This can facilitate early diagnosis and intervention, ultimately improving patient outcomes.

This study has some limitations. First, the sample size was small (n = 90). In addition, although this study reported that the number of turns during sleep is an important factor associated with PD, further studies are warranted to determine whether changes in the number of turns during sleep after exercise therapy and nutritional guidance affect the presence of PD. Second, as previously reported, the male participants in the present study turned more frequently during sleep than did the female participants. The decrease in sleep movements observed in this study could be attributed to akinesia and frequent awakenings (sleep disorders). These sleep disorders could be the result of conditions such as restless leg syndrome, periodic limb movements, parasomnias (rapid eye movement sleep behavior disorder), and sleep apnea. However, the effects of sleep problems on sleep movement were not evaluated in the present study. The decrease in sleep movements may also be associated with decreased daytime activity due to parkinsonism. Previous studies ^(28)^ have reported that gait function is the most reliable indicator of lower activity level in PD. Therefore, a full assessment of daytime activity level, particularly gait function, should have been conducted. Third, to rigorously quantify the number of turns, it is imperative to utilize instruments such as accelerometers rather than solely relying on visual observation. Finally, the severity of sarcopenia could not be assessed using grip strength or walking speed.

Future longitudinal studies are warranted to assess changes in the frequency of turns during sleep over time in patients with PD and its related disorders. This would allow for a better understanding of the progression of PD and its effects on sleep activity. Moreover, investigating the effects of various therapeutic interventions on sleep activity in patients with PD and its related disorders can facilitate the identification of effective treatment strategies for improving sleep and overall quality of life. Furthermore, studies that involve the analysis of a range of disease stages and include larger sample sizes and participants from diverse backgrounds could help generalize these findings and validate the cutoff number of turns during sleep as a diagnostic tool. In addition, the development of a device that can easily measure the number of turns during sleep is desirable. The association between PD and other sleep parameters can also be analyzed in future studies to provide a more comprehensive understanding of the effects of PD on sleep quality. Investigation of the underlying pathophysiological mechanisms that link PD and its related disorders to reduced sleep activity can facilitate the development of targeted therapies for mitigating sleep disturbances associated with these conditions.

In conclusion, this study reported that the number of turns during sleep is an important factor associated with the presence of PD and its related disorders. Also, this study reported that in patients with PD and its related disorders, the number of turns during sleep may decrease before patients present with sarcopenia. Furthermore, PD and its related disorders may coexist in men who turn less than nine times during sleep and in women who turn less than six times during sleep.

## Article Information

### Conflicts of Interest

None

### Acknowledgement

We thank Editage (www.editage.com) for writing support.

### Author Contributions

All authors meet the ICMJE authorship criteria. All authors contributed to the intellectual content of the manuscript. All authors contributed to the study conception and design. Material preparation, data collection, and analysis were performed by Tokuharu Tanaka, Hidenori Onishi, Masaki Kiyono, Yuki Miyazaki, Azusa Tanaka, Akihiko Tanizawa, Tadanori Hamano, Hiroyuki Hayashi, Koji Kobayashi, and Osamu Yamamura. The first draft of the manuscript was written by Tokuharu Tanaka, and all the authors commented on previous versions of the manuscript. All authors are responsible for the interpretation of data and agree to be accountable for all aspects of the work in ensuring that questions related to the accuracy or integrity of any part of the work are appropriately investigated and resolved. All authors critically revised the manuscript for important intellectual content and approved the final version.

### Approval by Institutional Review Board (IRB)

Approval for this study was reviewed and granted by the Ethics Committees of the University of Fukui Hospital and Sugita Genpaku Memorial Obama Hospital (Approval No.: 20180060, 30-2). This study was conducted in accordance with the Declaration of Helsinki. All researchers involved in this study complied with the Ethical Guidelines for Medical and Biological Research Involving Human Subjects (MEXT/MHLW/METI Notification No. 1 of March 23, 2021). Written informed consent was obtained from participants or their representatives. The study subjects were allowed to refuse to participate in the study or to withdraw their consent for participation at any time.

The name of the trial registry: UMIN Clinical Trials Registry

Clinical trial registration number: UMIN000052436

### Data Sharing

All data underlying the findings are within the paper.
